# Micronutrient Deficiencies and Nutritional Status in Children with Celiac Disease: A Retrospective Study

**DOI:** 10.3390/children13040547

**Published:** 2026-04-15

**Authors:** Demet Teker Düztaş, Mahmut Esat Tülüce, Gizem Özata Uyar

**Affiliations:** 1 Department of Pediatric Gastroenterology, Ankara Etlik City Hospital, 06170 Ankara, Turkey; demettduztas@gmail.com; 2 Department of Pediatric Gastroenterology, Şanlıurfa Training and Research Hospital, 63040 Şanlıurfa, Turkey; mtuluce@phcc.gov.qa; 3 Department of Nutrition and Dietetics, Faculty of Health Sciences, Kırıkkale University, 71450 Kırıkkale, Turkey

**Keywords:** celiac disease, micronutrients, malnutrition, tTG-IgA, gluten-free diet, stunting, children

## Abstract

**Highlights:**

**What are the main findings?**

- Iron deficiency anemia was the most common micronutrient deficiency in children with celiac disease.
- Folate deficiency and ferritin levels were significantly associated with serological activity and adherence to a gluten-free diet.

**What is the implication of the main finding?**

- Poor adherence to a gluten-free diet may negatively affect both serological response and linear growth in children with celiac disease.
- Comprehensive follow-up including growth monitoring and micronutrient evaluation is essential for long-term management.

**Abstract:**

Background and aim: Celiac disease (CD) is a systemic autoimmune disorder triggered by gluten ingestion, and the only effective treatment is strict adherence to a gluten-free diet (GFD). Many factors, including limited dietary diversity and poor adherence, are associated with an increased risk of specific micronutrient deficiencies and malnutrition. This study aims to evaluate the relationship between adherence to GFD, celiac antibody levels, micronutrient levels, and nutritional status in children with CD. Methods: This retrospective study was conducted on 402 children aged 2–18 years with a diagnosis of CD confirmed positive by anti-tTG IgA and duodenal biopsy, all of whom had been on GFD for at least six months. Demographic, anthropometric, clinical, serological, and biochemical data (including hemogram, serum iron, ferritin, vitamin D, folate, and B12 levels), and GFD adherence were collected from medical records. Results: Most individuals are girls (64.9%), with a mean age of 10.6 ± 4.20 years. Chronic malnutrition was observed in 29.4% of patients. Acute malnutrition was identified in 27.8% of children, and wasting was observed in 6.7%. Iron deficiency anemia was the most frequently encountered micronutrient deficiency among the patients (23.9%). The prevalence of stunting was significantly higher among individuals with positive tTG-IgA levels and poor adherence to the GFD. Conclusions: Poor adherence to the GFD and positive tTG-IgA levels were associated with higher rates of stunting, underlining the need for individualized dietary follow-up and regular monitoring of both nutritional status and serological response in children with CD.

## 1. Introduction

Celiac disease (CD) is a systemic immune-mediated disorder that primarily affects the small intestine and results from an abnormal immune response to gluten—a protein found in barley, wheat, and rye—in genetically predisposed individuals [1]. Although genetic, environmental, and immunological factors contribute to disease pathogenesis, gluten exposure remains the principal trigger [2]. Advances in diagnostic methods and increased awareness of the disease in recent years have led to a marked increase in the reported incidence of CD [3]. In Europe and the United States, the number of confirmed cases has risen two to fourfold over the past two decades [4]. 

At present, the only effective treatment for CD is a lifelong adherence to a gluten-free diet (GFD), which requires the complete elimination of all gluten-containing grains from the patient’s diet [5]. Poor adherence to a GFD may result in persistent intestinal inflammation and continued micronutrient malabsorption, particularly in the proximal small intestine. Strict adherence to a GFD is essential for symptom management, mucosal healing, rectifying vitamin and mineral deficiencies, and preventing long-term complications [6]. However, maintaining a strict GFD remains challenging for many patients due to various personal, social, and environmental factors. Moreover, the diet may not provide adequate and balanced nutrition, potentially leading to insufficient intake of several micronutrients [7]. In particular, GFDs may be relatively deficient in nutrients such as protein, folate, iron, niacin, riboflavin, thiamine, vitamin B12, zinc, selenium, and dietary fiber [8].

Children with CD during growth are at a high risk for nutritional deficiencies due to mucosal damage caused by gluten exposure in cases of poor adherence, as well as dietary restrictions even when adhering to the GFD [6,9,10]. Even with adherence to a GFD, the restrictive nature of the diet may be associated with inadequate intake of certain micronutrients, potentially resulting in new deficiencies over time [11,12]. Both scenarios may contribute to malnutrition and adversely affect growth and development in pediatric patients.

Despite its clinical importance, only a small number of studies in the literature have examined the effects of both scenarios on the nutritional and micronutrient status of children with CD [10,13]. Therefore, the primary aim of this study was to assess adherence to the GFD, tTG IgA levels as a marker of disease activity, and nutritional status in children with CD. The secondary aim was to examine how dietary adherence and tTG IgA status are associated with growth outcomes and micronutrient deficiencies.

## 2. Methods

### 2.1. Study Design

This retrospective cross-sectional study included patients aged 2–18 years with biopsy-confirmed CD who were followed at the Pediatric Gastroenterology Clinic of Şanlıurfa Training and Research Hospital between January 2023 and July 2024. Due to the retrospective nature of the study, the Institutional Review Board waived the requirement for informed consent from individual participants.

All patients presenting during the specified study period were screened using the ICD-10 code K90.0 to identify cases of CD. Sociodemographic characteristics, presenting symptoms, anthropometric measurements, comorbid conditions, physical examination findings, and laboratory data were obtained from the hospital’s electronic medical record system.

### 2.2. Study Population

**Figure 1:**
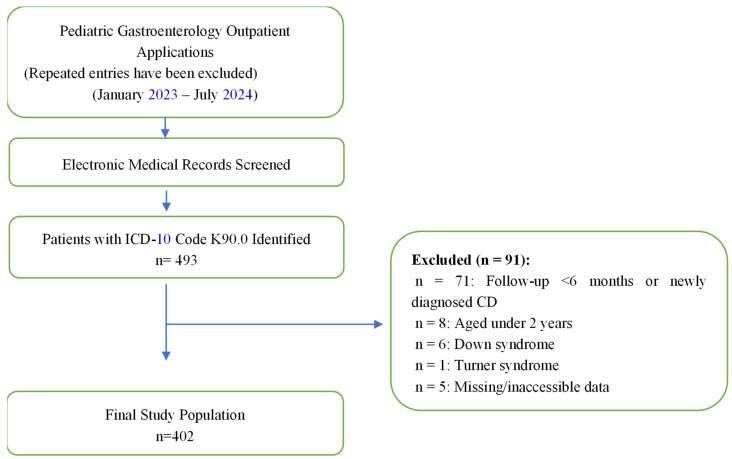
Flowchart of Patient Selection and Data Extraction.

Patients diagnosed with CD based on positive tissue transglutaminase IgA (tTG-IgA) levels and histological evidence of mucosal atrophy on duodenal biopsy, and who had been adhering to a gluten-free diet (GFD) for at least six months [14], were included in the study. In patients with selective IgA deficiency, anti-tTG-IgG levels were used for serological monitoring. All participants were evaluated by the same pediatric gastroenterologist. Exclusion criteria included inability to retrieve complete medical records, absence of biopsy-confirmed CD, presence of other chronic illnesses (e.g., renal diseases, cirrhosis, malignancies, organ failure), or genetic syndromes (e.g., Down syndrome, Turner syndrome). Details of the study population are shown in Figure 1.

### 2.3. Anthropometric Measurements

Height and weight measurements of all children included in the study were retrospectively obtained from previously recorded clinical data. These measurements had originally been performed according to conventional protocols, utilizing a mechanical scale and a stadiometer or altimeter, with the children attired in minimal or light clothing and without footwear. 

Nutritional status was evaluated by calculating z-scores for height for age (HFA), weight for age (WFA), weight for height (WFH), and body mass index for age (BMI for age) using WHO Anthro (version 3.2.2) and AnthroPlus (version 1.0.4) software. 

The age-based classification (<5 years and ≥5–18 years) was based on WHO growth assessment recommendations. Weight-for-height z-scores were used for children under five years of age, while BMI-for-age z-scores were used for children aged five years and older [15].

HFA z-scores were used to classify chronic malnutrition. Values below −2 standard deviations (SD) were defined as stunting, values between −2 and +2 SD as normal stature, and values above +2 SD as tall stature [15]. 

The age-based classification method is summarized in Table 1 [15]. Wasting was defined as a WFH or a BMI-for-age below −2 SD [15,16]. 

### 2.4. Clinical Characteristics and Symptoms

Demographic and anthropometric data of the patients were obtained from medical records. These records included information on age, sex, date of diagnosis, CD-related comorbidities (such as Type 1 Diabetes Mellitus, Hashimoto’s thyroiditis, vitiligo, etc.), clinical symptoms, body weight, height, biochemical and hematological evaluations, anti-tTG IgA levels (or anti-tTG IgG in cases of selective IgA deficiency), and information regarding adherence to the GFD.

### 2.5. Evaluation of Laboratory Results

Hemogram parameters and levels of serum iron, vitamin D, folic acid, and vitamin B12 were evaluated in participants diagnosed with CD. The relevant biochemical data were retrospectively obtained from routine clinical follow-up tests performed after initiation of the GFD.

Serum iron (µg/dL), 25-hydroxyvitamin D (25(OH)D) (ng/mL), folic acid (ng/mL), and vitamin B12 (pg/mL) levels were measured in the hospital’s central biochemistry laboratory using automated analyzer systems, in accordance with the manufacturers’ protocols. Routine internal quality control procedures of the clinical laboratory were applied to all tests.

Biochemical parameters were categorized by the laboratory as “deficiency,” “insufficiency,” or “within the normal range” according to established reference values. Anemia was defined according to World Health Organization (WHO) criteria for age: hemoglobin levels <11 g/dL for children aged 2–5 years, <11.5 g/dL for ages 5–11 years, <12 g/dL for boys and girls aged 12–14 years, <12 g/dL for females aged ≥15 years, and <13 g/dL for males aged ≥15 years [17]. Anemia was further classified morphologically using mean corpuscular volume (MCV) values as microcytic (<80 fL), normocytic (80–100 fL), or macrocytic (>100 fL). Additional evaluation included measurement of serum ferritin, vitamin B12, and folate levels to differentiate iron deficiency anemia from megaloblastic anemia. Serum ferritin was used as an indicator of iron stores, with levels < 15 μg/L considered indicative of depleted iron stores [18]. Folate deficiency was defined as serum folate levels < 4 nmol/L [19], and vitamin B12 deficiency as serum B12 levels < 203 pg/mL [19]. Vitamin D status was assessed based on serum 25(OH)D levels, with values < 20 ng/mL defined as deficiency, 20–30 ng/mL as insufficiency, and 30–50 ng/mL as sufficient [20]. 

All data were recorded by a single researcher using a standardized data collection form. To ensure data accuracy, a randomly selected subset of records was independently reviewed by a second researcher.

### 2.6. Evaluation of Serology

Serum anti-tTG IgA levels and, in cases with selective IgA deficiency, anti-tTG IgG levels were measured using commercially available Orgentec kits (Mainz, Germany) with the enzyme-linked immunosorbent assay (ELISA) method. All assays were performed according to the manufacturer’s instructions. The assay’s analytical measurement range was 20–200 U/mL; values outside this range were reported as the respective lower or upper limits. According to the manufacturer’s recommendations, a cut-off value of 20 U/mL was used to define seropositivity. 

Adherence to the gluten-free diet and anti-tTG IgA levels were assessed during the same clinical follow-up visit to ensure temporal alignment between these variables.

### 2.7. Assessment of Gluten-Free Diet Adherence 

The presenting symptoms of all participants were evaluated through a retrospective review of their medical records. Regardless of whether the symptoms were gastrointestinal or extraintestinal, individuals were classified as “symptomatic” or “asymptomatic” based on symptom presence.

Adherence to the GFD was retrospectively assessed from medical records using self-reported dietary information obtained during routine clinical visits. During these visits, patients and/or their families were asked about factors that could influence dietary adherence, including the feasibility of maintaining the diet in daily life, the frequency of intentional or accidental gluten exposure, eating habits at home and outside the home, label-reading practices, and social situations that might affect adherence.

Based on the reported information, patients who reported complete avoidance of gluten were categorized as having “very good” adherence. Those reporting occasional gluten intake were classified as having “occasional adherence,” whereas those reporting poor adherence to the GFD were categorized as having “very poor” adherence.

Clinical symptoms were obtained through retrospective review of medical records and may represent symptoms documented either at the time of diagnosis or during follow-up visits.

### 2.8. Statistical Analyses

Statistical analyses were performed using IBM SPSS Statistics for Windows, Version 25.0 (IBM Corp., Armonk, NY, USA). The continuous variable distribution was evaluated with the Kolmogorov–Smirnov test. The data were presented as mean ± standard deviation (mean ± SD), and categorical variables were presented as frequencies and percentages. 

Acute and chronic malnutrition status, micronutrient levels (iron, vitamin D, folic acid, and vitamin B12), and duration of GFD adherence according to GFD adherence status and anti-tTG IgA positivity/negativity were analyzed using the chi-square (χ^2^) test. A *p*-value of <0.05 was considered statistically significant for all analyses.

### 2.9. Ethical Approval and Data Protection

The study was approved by Ethics Committee of Harran University Clinical Research Center (dated 5 August 2024, approval number: HRU/24.11.17). Participants were informed that their data would be protected following confidentiality and data security principles. All patient information was recorded in the electronic database in an anonymized manner, and no personally identifiable data were used during the study. Only the research team had access to the password-protected system where the data was stored. The study was conducted in accordance with relevant legal regulations concerning personal data protection and the principles outlined in the Declaration of Helsinki.

## 3. Results

### 3.1. General Characteristics 

The general characteristics of patients with celiac disease are presented in Table 2. The study population was predominantly female. The mean age was 10.6 ± 4.20 years, while the mean age at diagnosis was 7.0 ± 3.52 years. In terms of adherence to the gluten-free diet, only approximately half of the participants demonstrated very good adherence.

### 3.2. Anthropometric Characteristics and Nutritional Status

**Figure 2:**
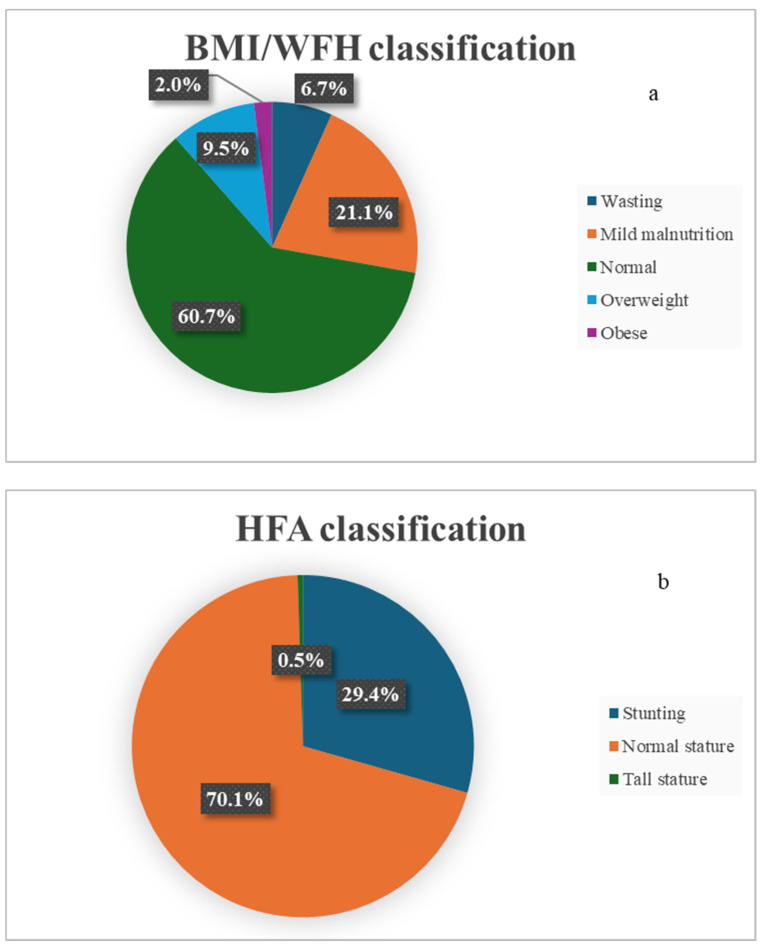
(**a**,**b**). Distribution of HFA and WFH/BMI classifications.

The distribution of BMI/WFH and HFA classifications is presented in Table 3 and Figure 2. The distribution of HFA, an indicator of chronic malnutrition, indicated that nearly one-third of the participants were classified as stunted, while the overall prevalence of acute malnutrition was 27.8%.

### 3.3. Clinical Symptoms and Comorbid Conditions

The clinical symptoms and comorbid conditions observed in children with CD are summarized in Table 4. Abdominal pain was the most commonly reported symptom, followed by diarrhea, abdominal distension, and loss of appetite. Type 1 diabetes mellitus was the most common comorbid condition, while other conditions, including autoimmune thyroiditis and selective IgA deficiency, were relatively infrequent (Table 4).

### 3.4. Micronutrient Status and Serological Findings

Micronutrient status and tTG-IgA serology of the participants are summarized in Table 5. Iron deficiency anemia and low ferritin were the most frequent micronutrient abnormalities. Vitamin D deficiency or insufficiency was present in the majority of patients (82.8%). Folate deficiency and vitamin B12 deficiency were less common.

### 3.5. Nutritional Characteristics According to tTG-IgA and GFD Status

Nutrition-related characteristics of the participants according to tTG-IgA status and adherence to a gluten-free diet are presented in Table 6. Patients with positive tTG-IgA more frequently exhibited short stature, shorter duration of gluten-free diet adherence, and poorer dietary compliance compared with those with negative tTG-IgA (*p* < 0.05). In addition, indicators of micronutrient deficiency, particularly low ferritin levels, folate deficiency, and iron deficiency anemia, were more prevalent in the tTG-IgA-positive group. No significant differences were observed between the groups in BMI/WFH classification, vitamin D, or vitamin B12 status (*p* > 0.05).

When evaluated according to dietary adherence, poorer adherence to the GFD was associated with a higher prevalence of stunting and increased rates of vitamin D deficiency and folate deficiency (*p* < 0.05).

## 4. Discussion

This study showed that evaluating anti-tTG IgA positivity alongside adherence to the GFD in children with CD provides important insights into their nutritional status and micronutrient profiles. In our study, approximately half of the participants reported good adherence to the GFD; however, anti-tTG-IgA positivity was still observed in a substantial proportion of these patients. Previous studies have reported GFD adherence rates ranging from 59% to 95%, which are comparable to the adherence rate observed in our study (67.7%) [21,22]. The persistence of serological positivity despite self-reported good adherence suggests ongoing gluten exposure through various mechanisms, including inadequate label-reading practices, hidden gluten in processed foods, and cross-contamination. It should also be noted that serological normalization after the initiation of the GFD may take longer than one year in some pediatric patients; therefore, persistent anti-tTG IgA positivity during follow-up may not necessarily indicate poor dietary adherence but may also reflect the duration of treatment. In this context, families’ knowledge and daily dietary practices play an important role in determining children’s adherence to the GFD. 

Implementing a GFD presents several challenges, including the limited nutritional quality of commercially available gluten-free products, difficulties in accessing these products, and the risk of gluten cross-contamination [21]. Many of these products are low in dietary fiber and certain micronutrients and are characterized by higher fat content, increased glycemic index, and glycemic load [23,24,25], which may contribute to micronutrient deficiencies even among patients who report good adherence [26,27]. Given that commercial gluten-free products constitute a substantial proportion of children’s daily energy intake [11,28], individualized dietary plans in which these products have a more limited role are essential for ensuring nutritional adequacy.

In children with CD, appropriate adherence to the GFD is generally expected to support normal weight gain and linear growth during the prepubertal period [14]. The European Society for Paediatric Gastroenterology, Hepatology and Nutrition (ESPGHAN) recommends monitoring anti-tTG-IgA as an indirect indicator of mucosal healing [14]. However, previous studies have shown that linear growth may not normalize in parallel with weight gain or tTG-IgA normalization [29,30,31]. This discrepancy has been attributed to reduced levels of insulin-like growth factor-1 (IGF-1) and growth hormone (GH) associated with CD [32,33]. In our study, a significant association was observed between tTG-IgA status and GFD adherence, with stunting being notably more prevalent among patients with unfavorable findings in both parameters.

Growth monitoring should therefore include both BMI and height z-scores [34]. The prevalence of overweight/obesity in our study population (11.5%) is consistent with previous studies [35,36]. Evidence from large national cohorts and meta-analyses further indicates that the majority of children with newly diagnosed celiac disease have a normal BMI (approximately 70–77%), with overweight/obesity rates ranging from about 10% to 18% [37,38,39]. Notably, these estimates vary according to geographic region and socioeconomic context, including differences in income level [38]. Taken together, these findings indicate that celiac disease in children cannot be excluded based solely on overweight or obesity status. In our study population, no significant association was observed between BMI/WFH classification and tTG-IgA status or adherence to a gluten-free diet, consistent with previous reports [40,41]. This suggests that BMI-based indicators may not adequately reflect overall nutritional status and may obscure impairments in linear growth. Accordingly, comprehensive assessment including height and z-scores should also be considered. 

ESPGHAN recommends assessing micronutrient levels at diagnosis and monitoring them until normal values are achieved [14], although clear guidance on optimal follow-up frequency remains limited. Micronutrient deficiencies may persist even after histological recovery [7], likely reflecting a combination of malabsorption and the restrictive nature of the gluten-free diet.

Adherence to the GFD is associated with a reduced risk of several micronutrient deficiencies compared with untreated celiac disease [42]. However, vitamin D insufficiency remains highly prevalent. In our study, 25(OH)D levels were within the normal range in only 17.2% of patients, consistent with previously reported rates ranging from 2.5% to 33.3% [43,44]. Better adherence to a gluten-free diet was associated with lower rates of deficiency. However, vitamin D status is also influenced by non-dietary factors such as geographical location and sun exposure [45], and optimal levels may not always be achieved even with supplementation [46]. These factors should be considered when interpreting vitamin D status in children with celiac disease.

Anemia is common in children with celiac disease, with reported prevalence rates ranging from 12% to 33% in untreated patients [43,47,48,49,50,51]. In our study, anemia prevalence (23.9%) was comparable. Ferritin was associated with tTG-IgA positivity, while folate deficiency was linked to both serological status and dietary adherence, suggesting ongoing inflammation or suboptimal adherence. In contrast, vitamin B12 deficiency was rare in our study and was not associated with serological status or dietary adherence, consistent with previous findings [9]. 

Long-term monitoring is essential, as adherence to a GFD has been shown to decline over time [52], with studies reporting that up to 61% of patients discontinue regular follow-up within five years [13]. In our cohort, the persistence of tTG-IgA positivity in a substantial proportion of patients on GFD for more than one year further highlights the need for structured, sustainable long-term follow-up in pediatric CD.

Strengths of our study include the large sample size, the multidimensional evaluation of adherence to the GFD using both serological markers and patient self-reports, and the comprehensive assessment of micronutrient deficiencies across a broad range of parameters. This integrated approach provides clinically relevant insights based on real-world data.

However, several limitations should be acknowledged. First, due to the retrospective design, data on dietary supplement use was unavailable, representing a potential confounder in the evaluation of micronutrient levels. Second, the lack of information on family history of short stature limited the ability to account for genetic influences on growth. Third, detailed dietary intake data were not available, precluding differentiation between malabsorption and inadequate intake as causes of micronutrient deficiencies. Fourth, baseline micronutrient measurements at diagnosis were not consistently available, limiting longitudinal evaluation. Fifth, adherence to the gluten-free diet was assessed retrospectively based on self-report, which may introduce reporting bias; future studies should incorporate standardized dietary assessments or objective biomarkers. Sixth, potential confounders such as socioeconomic factors and sun exposure were not captured. Seventh, differences in screening practices for autoimmune comorbidities may have introduced detection bias, potentially leading to underestimation of autoimmune thyroiditis compared with type 1 diabetes mellitus. Finally, the lack of histological follow-up limits direct confirmation of mucosal healing, although this approach is consistent with current ESPGHAN recommendations.

Overall, our findings suggest that micronutrient and nutritional deficiencies may persist not only at the time of diagnosis but also during the follow-up period in children with CD. Nevertheless, it remains unclear whether these deficiencies are primarily related to persistent intestinal malabsorption associated with inadequate adherence to the GFD or to insufficient and unbalanced nutrient intake associated with the restrictive nature of the diet. These observations highlight the importance of evaluating micronutrient deficiencies not only from a biological perspective but also within the broader context of dietary behaviors and nutritional practices.

## 5. Conclusions

This study identified iron deficiency anemia as the most prevalent micronutrient deficiency in children with CD. Folate deficiency was significantly associated with both tTG-IgA levels and adherence to the GFD, whereas ferritin levels were significantly associated with tTG-IgA positivity, a marker of serological activity. Furthermore, a significant relationship was observed between tTG-IgA levels and GFD adherence, with markedly higher rates of stunting among patients showing unfavorable findings in both parameters. These findings suggest that poor dietary adherence may be associated with unfavorable serological response and impaired linear growth. Overall, our results underscore the importance of evaluating both biochemical markers and growth parameters during the long-term follow-up of children with CD.

From the time of diagnosis, patients should receive individualized nutritional counseling that emphasizes the consumption of naturally gluten-free foods, improves understanding of commercial product labeling, and ensures appropriate energy, macronutrient, and micronutrient balance. These strategies may be incorporated into routine clinical follow-up protocols to support the prevention and early detection of malnutrition and micronutrient deficiencies. Furthermore, prospective, multicenter studies with structured follow-up are needed to better evaluate the interplay between GFD adherence, micronutrient status, and long-term health outcomes.

## Figures and Tables

**Table 1:** Approach to the Evaluation of Acute Malnutrition [15].

BMI/WFH z Score		
	<5 age	≥5–18 age
Severe malnutrition	<−3	<−3
Moderate malnutrition	−2 to −2.9	−2 to −2.9
Mild malnutrition	−1 to −1.9 z score	−1 to −1.9 z score
Normal	−1 to 1.9	−1 to 1.0
Overweight	2.0 to 3.0	>1.0 to 2.0
Obese	≥3.0	≥2

**Table 2:** General characteristics in children with CD (*n*: 402).

	*n* (%) or Mean ± SD
Gender	
Girls	261 (64.9)
Boys	141 (35.1)
Age (years)	10.6 ± 4.20
Age groups	
2–6	66 (16.4)
7–11	175 (43.5)
≥12	161 (40.1)
Age at diagnosis of CD	7.0 ± 3.52
Duration of diagnosis (years)	3.6 ± 3.05
Weight (kg)	32.3 ± 14.84
WFA z score *	−0.9 ± 1.11
Height (cm)	132.6 ± 21.29
HFA z score	−1.3 ± 1.16
BMI (kg/m^2^)	17.6 ± 7.31
BMI for age z score	−0.3 ± 1.19
Use of enteral support products	
Yes	6 (1.5)
No	396 (98.5)
Duration of gluten-free diet in years	
<1 year	64 (15.9)
≥1 years	338 (84.1)
Adherence to gluten-free diet	
Very good	212 (52.7)
Occasional adherence	121 (30.1)
Very poor	69 (17.2)

* These values are for individuals aged 2 to 10 years. HFA: height for age, BMI: body mass index, WFA: weight for age.

**Table 3:** Distribution of HFA and BMI/WFH classifications among children with celiac disease.

	*n* (%)
HFA classification	
Stunting	118 (29.4)
Normal stature	282 (70.1)
Tall stature	2 (0.5)
BMI/WFH classification	
Severe malnutrition	6 (1.5)
Moderate malnutrition	21 (5.2)
Mild malnutrition	85 (21.1)
Normal	244 (60.7)
Overweight	38 (9.5)
Obese	8 (2.0)

HFA: height for age, BMI: body mass index, WFH: weight for height.

**Table 4:** The clinical symptoms and comorbid conditions in children with CD.

Clinical Symptom	*n* (%)	Comorbid Conditions	*n* (%)
Abdominal pain	74 (18.4)	Tip 1 diabetes mellitus	27 (6.7)
Diarrhea	33 (8.2)	Autoimmune thyroiditis	4 (0.9)
Abdominal distension	26 (6.5)	Selective IgA deficiency	4 (1.0)
Constipation	16 (4.0)	Vitiligo	1 (0.2)
Nausea/vomiting	10 (2.5)		
Loss of appetite	23 (5.7)		
Irregular menstruation	15 (3.7)		
Bone pain	2 (0.5)		

**Table 5:** Micronutrient status, anemia, and tTG-IgA status among children with CD.

Clinical Symptom	*n* (%)
Iron deficiency anemia	
Yes	96 (23.9)
No	306 (76.1)
Ferritin	
Low ferritin	149 (37.1)
Normal ferritin	253 (62.9)
Serum vitamin B12	
Deficiency	19 (4.7)
Normal	383 (95.3)
Serum vitamin D	
Deficiency	209 (52.0)
Insufficiency	124 (30.8)
Sufficiency	69 (17.2)
Folate	
Deficiency	42 (10.4)
Normal	360 (89.6)
tTG IgA	
Negative	130 (32.3)
Positive	272 (67.7)

**Table 6:** Participants’ nutrition-related characteristics according to tTG IgA and adherence to gluten-free diet *n* (%).

	tTG IgA	tTG IgA		Adherence to Gluten-Free Diet			
	Negative (*n* = 130)	Positive (*n* = 272)	*p*-Value	Very Good (*n* = 212)	Occasional Adherence (*n* = 121)	Very Poor (*n* = 69)	*p*-Value
Height for age							
Stunting	28 (21.5)	90 (33.1)	X^2^ = 5.115	62 (29.2)	27 (22.3)	29 (42.0)	X^2^ = 8.239
Normal/tall stature	102 (78.5)	182 (66.9)	*p* = 0.024 *	150 (70.8)	94 (77.7)	40 (58.0)	*p* = 0.016 *
BMI/WFH z score							
Wasting	8 (6.2)	19 (7.0)	X^2^ = 0.097	10 (4.7)	10 (8.3)	7 (10.1)	X^2^ = 3.451
Normal	107 (82.3)	222 (81.6)	*p* = 0.953	178 (84.0)	98 (81.0)	53 (76.9)	*p* = 0.485
Overweight/obese	15 (11.5)	31 (11.4)		24 (11.33)	13 (10.7)	9 (13.0)	
Duration of gluten-free diet							
<1 year	12 (9.2)	52 (19.1)	X^2^ = 6.423	42 (19.8)	14 (11.6)	8 (11.6)	X^2^ = 5.073
≥1 years	118 (90.8)	220 (80.9)	*p* = 0.011 *	170 (80.2)	107 (88.4)	61 (88.4)	*p* = 0.079
Adherence to gluten-free diet							
Very good	86 (66.2)	126 (46.3)	X^2^ = 22.263				
Occasional adherence	37 (28.5)	84 (30.9)	*p* < 0.001 *				
Very poor	7 (5.4)	62 (22.8)					
Iron deficiency anemia							
Yes	21 (16.2)	75 (27.6)	X^2^ = 6.310	51 (24.1)	30 (24.8)	15 (21.7)	X^2^ = 0.233
No	109 (83.8)	197 (72.4)	*p* = 0.012 *	161 (75.9)	91 (75.2)	54 (78.3)	*p* = 0.890
Ferritin							
Low	32 (24.6)	117 (43.0)	X^2^ = 12.765	80 (37.7)	40 (33.1)	29 (42.0)	X^2^ = 1.603
Normal	98 (75.4)	155 (57.0)	*p* < 0.001 *	132 (62.3)	81 (66.9)	40 (58.0)	*p* = 0.449
Serum vitamin B12							
Deficiency	3 (2.3)	16 (5.9)	X^2^ = 2.496	7 (3.3)	7 (5.8)	5 (7.2)	X^2^ = 2.230
Normal	127 (97.7)	256 (94.1)	*p* = 0.114	205 (96.7)	114 (29.8)	64 (16.7)	*p* = 0.328
Serum vitamin D							
Deficiency	57 (43.8)	152 (55.9)	X^2^ = 5.106	95 (44.8)	69 (57.0)	45 (65.2)	X^2^ = 10.508
Insufficiency	47 (36.2)	77 (28.3)	*p* = 0.078	76 (35.8)	33 (27.3)	15 (21.7)	*p* = 0.033 *
Sufficiency	26 (20.0)	43 (15.8)		41 (19.3)	19 (15.7)	9 (13.0)	
Folat							
Deficiency	5 (3.8)	37 (13.6)	X^2^ = 8.950	10 (4.7)	17 (14.0)	15 (21.7)	X^2^ = 18.552
Normal	125 (96.2)	235 (86.4)	*p* = 0.003 *	202 (95.3)	104 (86.0)	54 (78.3)	*p* < 0.001 *

* *p* < 0.05 statistically significant.

## Data Availability

The data may be available from the corresponding author upon reasonable request.

## Abbreviations

BMI	Bosy mass index
CD	Celiac disease
ESPGHAN	European Society for Paediatric Gastroenterology, Hepatology and Nutrition
GFD	Gluten-free diet
MUAC	Mid-upper arm circumference
tTG-IgA	Tissue Transglutaminase Immunoglobulin A
WFA	Weight for age
WFH	Weight for height
HFA	Height for age
